# Determinants of Maternal Diet Quality in Winter in the Kyrgyz Republic

**DOI:** 10.9745/GHSP-D-21-00720

**Published:** 2022-12-21

**Authors:** Altrena Mukuria-Ashe, Silvia Alayon, Tim Williams, Gulshat Sydykova, Disha Ali, Erin Milner

**Affiliations:** a USAID Advancing Nutrition, Arlington, VA, USA.; b Save the Children USA, Washington, DC, USA.; c Formerly of Strengthening Partnerships, Results, and Innovations in Nutrition Globally, Washington, DC, USA.; d Independent consultant, Addis Ababa, Ethiopia; Formerly of John Snow, Inc.; e Independent consultant; Formerly of Strengthening Partnerships, Results, and Innovations in Nutrition Globally, Bishkek, Kyrgyz Republic.; f Public Health Institute/USAID Sustaining Technical and Analytical Resources, Washington, DC, USA.

## Abstract

Diet quality influences maternal health and nutrition from preconception through pregnancy and lactation, as well as infant health and nutrition. Women are vulnerable to poor nutrition given their increased nutrient requirements during the prenatal period through the first 2 years postpartum. Minimum diet diversity among women, a good predictor of adequate micronutrient intake, is often used as a proxy for diet quality. The Kyrgyz Republic is experiencing a triple burden of malnutrition—stunting in children, overweight and obesity in women, and micronutrient deficiencies and anemia in both women and children. In this study, we assessed factors associated with the quality of maternal diets in winter when micronutrient-rich foods may be more difficult to access due to scarcity and price. We conducted secondary data analysis from a survey conducted in the winter of 1,359 mothers of children aged younger than 2 years. Women were asked about the types of foods they stored and preserved in the fall and whether any remained in winter. After controlling for maternal characteristics, household size, main source of income, and region (including urban and rural), women with preserved food remaining at the time of the survey, who stored more than 4 different types of food in the fall, and who lived in Jalal-Abad oblast were more likely to have consumed a minimally diverse diet. Where seasonality affects food availability, promoting culturally appropriate home processing of a variety of foods in the fall and increased market access may improve diet diversity in winter.

## Abstract (precis)

Contrary to cultural dietary preferences, mothers in the Kyrgyz Republic maintained minimum diet diversity during winter through access to markets and by growing, preserving, and storing foods.

## Teaser

**Key Findings:**

- Mothers in the Kyrgyz Republic can consume a diverse diet in winter despite food accessibility challenges.
- Diet diversity was strongly associated with cultural practices of preserving and storing foods in the fall season.

**Key Implications:**

- Program managers should identify positive cultural practices that can be promoted to support improved diet diversity.
- Policy makers and program managers should engage food systems and market access as part of any intervention to improve diets.

## INTRODUCTION

The quality of maternal diets influences both maternal and infant health and nutrition. Good maternal nutrition before and during pregnancy is associated with reduced risk of maternal mortality, preterm birth, low birthweight, and child stunting.^1^^–^^3^ However, pregnant and lactating women in low-and middle-income countries rarely consume sufficient amounts of vegetables, fruit, meat, and dairy to meet their nutritional needs.^4^^,^^5^ Multiple factors influence maternal diets, including cultural beliefs, personal preferences and needs, knowledge, food availability, and household food security, among others.^5^^,^^6^

Diet diversity—a component of diet quality—is often used as a proxy for micronutrient adequacy. Based on the number of food groups consumed over the past 24 hours, minimum diet diversity for women (MDD-W) is a good predictor of the adequacy of micronutrient intake for women aged 15 to 49 years.^7^^–^^9^ Strategies often used to diversify diets include increasing household production of nutrient-dense foods, market accessibility and availability of micronutrient-rich foods, and nutrition education to increase demand and consumption of nutrient-dense foods, making iron-, zinc-, and vitamin A–rich foods more available.^10^^–^^12^ In the Kyrgyz Republic, produce is usually preserved or stored for the long winter months but often consumed before spring, potentially leaving families without foods rich in vitamin A, vitamin C, folate, and iron.^13^ Little has been published on the impact of winter on diets in low-and middle-income countries. In Pakistan, a study found that dietary diversity increased in winter due to storage and market purchases.^14^ Whereas in Iran, a study found that the consumption of fruits and vegetables declined during the cold seasons.^15^

## MALNUTRITION IN THE KYRGYZ REPUBLIC

The Kyrgyz Republic is a landlocked country in Central Asia that covers 199,000 square kilometers (76,834 square miles) and borders China, Kazakhstan, Uzbekistan, and Tajikistan. Its terrain is primarily mountainous with deep fertile valleys; although the climate is considered continental with cold winters and hot summers, it varies with altitude.^16^ The climate in the Kyrgyz Republic includes 4 seasons: spring (March–May); summer (May–September); fall (September–December); and winter (December–February). The mountainous terrain and harsh winter climate create additional challenges in accessing food and water for nearly half the year. The prevalence of moderate to severe food insecurity in the total population is 23.9%.^17^

The majority of the country’s multiethnic population of more than 6 million people live in rural areas, and there is a considerable gap in the standard of living between these families and those in the major urban centers.^13^ The World Food Programme reported in 2020 that 22.4% of the population lives below the poverty line.^17^ In the Kyrgyz Republic, diets primarily consist of meats and starches.^18^ For much of the rural population, herding animals is their main livelihood source. The main food crops grown include wheat, barley, maize, potatoes, vegetables, and fruits. Sugar beet, cotton, and tobacco are important cash crops. Fresh fruit and vegetable consumption is limited to summer, and in winter, only produce that can be stored for long periods of time, such as apples and pears, is consumed.^19^

The Kyrgyz Republic has a triple burden of malnutrition, where overweight or obesity and undernutrition exist in the same communities as micronutrient deficiencies. Almost 12% of children aged younger than 5 years are stunted (low height-for-age), 43% of children aged 6–59 months are anemic, 36% of women of reproductive age are overweight or obese (body mass index ≥25), and 35% of women of reproductive age are anemic.^20^^–^^22^ Globally, dietary intake is a major determinant of malnutrition (both undernutrition and overweight or obesity); improved diets provide an opportunity to improve the nutrition situation.^23^^,^^24^

The Kyrgyz Republic has a triple burden of malnutrition, where overweight or obesity and undernutrition exist in the same communities as micronutrient deficiencies.

### SPRING Project

Strengthening Partnerships, Results, and Innovations in Nutrition Globally (SPRING) was a 7-year U.S. Agency for International Development (USAID)–funded project to strengthen global and country efforts to scale up high-impact nutrition practices and policies and improve maternal and child nutrition outcomes. The project was managed by JSI Research & Training Institute, Inc., with partners Helen Keller International, The Manoff Group, Save the Children, and the International Food Policy Institute.^25^ In collaboration with the government of the Kyrgyz Republic, from 2014 to 2018, SPRING sought to improve the nutritional status of children aged younger than 2 years and women of reproductive age through the uptake of 11 evidence-based nutrition-related practices. These practices, tailored to the Kyrgyz context, included optimal breastfeeding, appropriate complementary feeding of children, dietary diversity throughout the year, reduction of junk food, handwashing, and other household-level behaviors targeting women and children in the first 1,000 days. SPRING’s geographic focus was 6 rayons and townships in Naryn oblast and 11 rayons and townships of Jalal-Abad oblast within the USAID Feed the Future zone of influence—targeted geographic areas in 19 focus countries where USAID works jointly with host governments and other partners to address global hunger and food insecurity by supporting agriculture sector growth and improving the nutritional status of the population. At the national level, SPRING supported the development of 2 clinical protocols on anemia and deworming.^13^

The objective of this study is to explore the determinants of the diversity of maternal diets in winter when dietary diversity may be low because micronutrient-rich foods are not easily accessible and infrequently consumed due to cultural preferences, scarcity, and price.^13^^,^^19^ We specifically looked at the consumption of food groups rich in vitamin A, iron, and zinc promoted by SPRING and the government to increase diet diversity. We asked the following research questions: (1) Which food groups are consumed by mothers of children aged younger than 2 years in winter in select areas of the Kyrgyz Republic? (2) What factors are associated with maternal dietary diversity in winter when the availability and accessibility of diverse foods are limited?

## METHODS

We conducted secondary data analyses of an endline survey that was part of the evaluation of SPRING in the Kyrgyz Republic.^13^ SPRING’s activities targeted traditionally underserved populations including those living in remote and mountainous regions within the Feed the Future zone of influence.^13^ The cross-sectional endline survey was conducted in selected villages and towns in 1 rayon (Jumgal) in Naryn oblast, 1 rayon (Uzgen) in Osh oblast, and 4 rayons within Jalal-Abad oblast, including periurban and rural communities. SPRING was only active in the rayons in Naryn and Jalal-Abad oblasts. The mostly rural Uzgen rayon in Osh oblast was included as a comparison area in the survey because its geographic, ethnic, and climatic characteristics are roughly in between those of Jalal-Abad and Jumgal. A total of 1,359 mothers of children aged 0–23 months were interviewed in late winter (February–March 2017) representing Jalal-Abad (455), Jumgal (453), and Uzgen (451).

A multistage cluster sampling approach was applied with villages/towns as the primary sampling unit, using probability proportion to size. From each primary sampling unit, using simple random sampling, 15–30 households with mothers of children aged 0–23 months were selected. Details of the sampling method can be found in the endline report.^26^

Using an open, list-based recall of the foods consumed in the previous 24 hours, the survey collected data on the dietary practices of women and their children aged 6–23 months. Given differences in topography, sources of food were expected to differ among rayons. To understand how this influences intake, the survey included questions about the sources from which different types of food were obtained (e.g., farm, market, or homegrown). The survey also asked whether households preserved or stored key food items such as grains, vitamin A–rich foods, and other vegetables and fruits (e.g., cabbage, onions, eggplant, cucumbers, apples, persimmons, and melon) in the fall, and whether any stored/preserved food items remained at the time of the survey.

The main outcome of interest was MDD-W—consuming at least 5 of 10 standardized food groups.^9^ We hypothesized several factors would be associated with MDD-W in winter: having access to a smallholding farm or garden, storing or preserving food in the fall, the diversity of food items that were stored in the fall for winter consumption, food remaining from storage or preservation at the time of the survey, and access to local markets. SPRING and the Government of the Kyrgyz Republic selected these cultural practices (food storage and preservation in the fall) to promote the consumption of nutrient-dense foods.

Food storage and preservation in fall were practices selected by SPRING and the government to promote consumption of nutrient-dense foods.

The data were analyzed using Stata version 15 (StataCorp). Analyses were conducted using “svy” commands to account for the multistage survey design. The dependent variable was consumption of minimum dietary diversity (5 food groups or more) versus those who consumed less.

The associations between the MDD-W and the variables of interest were examined using bivariate analysis and chi-square test to determine the significance of association. Explanatory variables were further tested while controlling for potential confounders using logistic regression.

## RESULTS

### Study Sample

Table 1 shows the background characteristics of the sample of women from the 3 urban and rural areas. Their mean ages were similar across areas, varying from 27.3 to 29.6 years. The mean household size is about 6 members. The majority of the women completed at least 10–11 years of education, and the proportion of women with some postsecondary education ranges from 22% to 31%. The main source of household income varies between areas. In Jalal-Abad the predominant sources of income were business/commerce/professional (32.7%), whereas in Uzgen the main source of income was agriculture (50.8%); in Jumgal, 34.7% of households derived their incomes from other unknown sources. The majority of women in Jumgal (66.4%) and Uzgen (55.0%) reported having access to homegrown foods. A smaller proportion of women in Jalal-Abad (44.4%) reported access to homegrown foods. The majority of women in all 3 areas consumed 5 or more food groups.

**TABLE 1:** Sociodemographic Characteristics of Mothers of Children Aged Younger Than 2 Years, by Region, Kyrgyz Republic

	**Jalal-Abad (n=455)**	**Jumgal (n=453)**	**Uzgen (n=451)**
Age, years, mean (SD)	28.4 (5.8)	29.6 (6.1)	27.3 (5.3)
Household size, mean (SD)	5.7 (1.9)	6.2 (1.6)	6.3 (2.3)
Food groups consumed, mean (SD)	5.8 (1.8)	5.2 (1.7)	5.3 (1.7)
Consumed at least 5 of 10 food groups, %	76.0	62.4	68.1
Ethnicity, %			
Kyrgyz	79.6	99.3	82.9
Uzbek	19.3	0	3.6
Tatar/Russian	1.1	0.7	13.5
Urban/rural residence, %			
Rural	53.0	100	100
Urban	47.0	0	0
Education level, %			
9 years or less (less than secondary school)	6.4	4.9	7.8
10–11 years (some secondary school)	51.4	56.3	57.4
Vocational/technical or some higher education	11.0	9.7	12.4
Some postsecondary education	31.2	29.1	22.4
Main sources of household income, %			
Agriculture (raising livestock or crops)	20.3	29.1	50.8
Laborers/migrant workers	29.1	14.0	24.3
Business/commerce/salaried professionals	32.7	22.2	18.5
Others	17.9	34.7	6.5
Access to homegrown foods, %	44.4	66.4	55.0

Abbreviation: SD, standard deviation.

### Food Groups Consumed

Of the 10 food groups, almost all mothers in all 3 geographical areas consumed grains, roots, and tubers (staple foods). More than 90% consumed meat, poultry, and fish, and most consumed dairy (70.3%–80.7%) in the 24 hours before surveying. The food groups consumed by the smallest proportion of mothers were nuts and seeds (in Jalal-Abad, 21.8%; Uzgen, 21.5%; Jumgal, 12.8%), other vegetables (in Jalal-Abad, 21.1%; Uzgen, 10.2%), and pulses (legumes) (in Jalal-Abad, 14.5%; Jumgal, 13.3%; Uzgen, 11.1%). In Jumgal, some mothers (19%) consumed eggs (Table 2).

**TABLE 2.** Percentage of Mothers of Children Aged Younger Than 2 Years Who Consumed Each of the 10 Food Groups, by Region, Kyrgyz Republic

**Food Group**	Jalal-Abad, %(95% CI)	Jumgal, %(95% CI)	Uzgen, %(95% CI)
Dairy	80.7 (70.8, 87.7)	90.9 (88.7, 92.7)	70.3 (64.6, 75.4)
Grains, roots, tubers	100	99.5 (97.9, 99.9)	99.7 (98.1, 100)
Meat, poultry, fish	93.6 (89.0, 96.3)	91.6 (87.2, 94.6)	91.5 (88.5, 93.8)
Eggs	45.7 (40.1, 51.3)	18.9 (13.5, 26.1)	42.3 (35.8, 49.1)
Nuts and seeds	21.7 (14.8, 30.7)	12.8 (8.7, 18.3)	21.5 (16.2, 28.0)
Pulses (legumes)	14.5 (10.3, 20.0)	13.2 (9.8, 17.6)	11.1 (7.9, 15.2)
Vitamin A–rich fruits and vegetables	79.9 (74.7, 84.0)	63.3 (57.3, 69.0)	72.9 (66.7, 78.3)
Dark green leafy vegetables	47.0 (41.7, 52.4)	34.2 (27.3, 41.8)	43.0 (34.7, 51.6)
Other vegetables^a^	21.1 (14.8, 29.1)	24.9 (18.2, 33.2)	10.2 (7.2, 14.2)
Other fruits^b^	76.0 (67.1, 83.1)	70.8 (65.9, 75.3)	67.2 (61.6, 72.2)

Abbreviation: CI, confidence interval.

a Such as cabbage, onions, eggplant, and cucumbers.

b Such as apples, pears, persimmons, and melon.

### Consumption of Micronutrient-Rich Foods

We examined the consumption of food groups rich in vitamin A, iron, and zinc promoted by SPRING and the government to increase diet diversity. It is not surprising that the iron-rich and zinc-rich food groups are high considering the high percentage of mothers consuming meat. Since fresh vitamin A–rich produce and eggs may be harder to access, foods rich in vitamin A are consumed by a smaller percentage of mothers in winter (Figure).

Since fresh vitamin A–rich produce and eggs may be harder to access, foods rich in vitamin A are consumed by a smaller percentage of mothers in winter.

**FIGURE.**
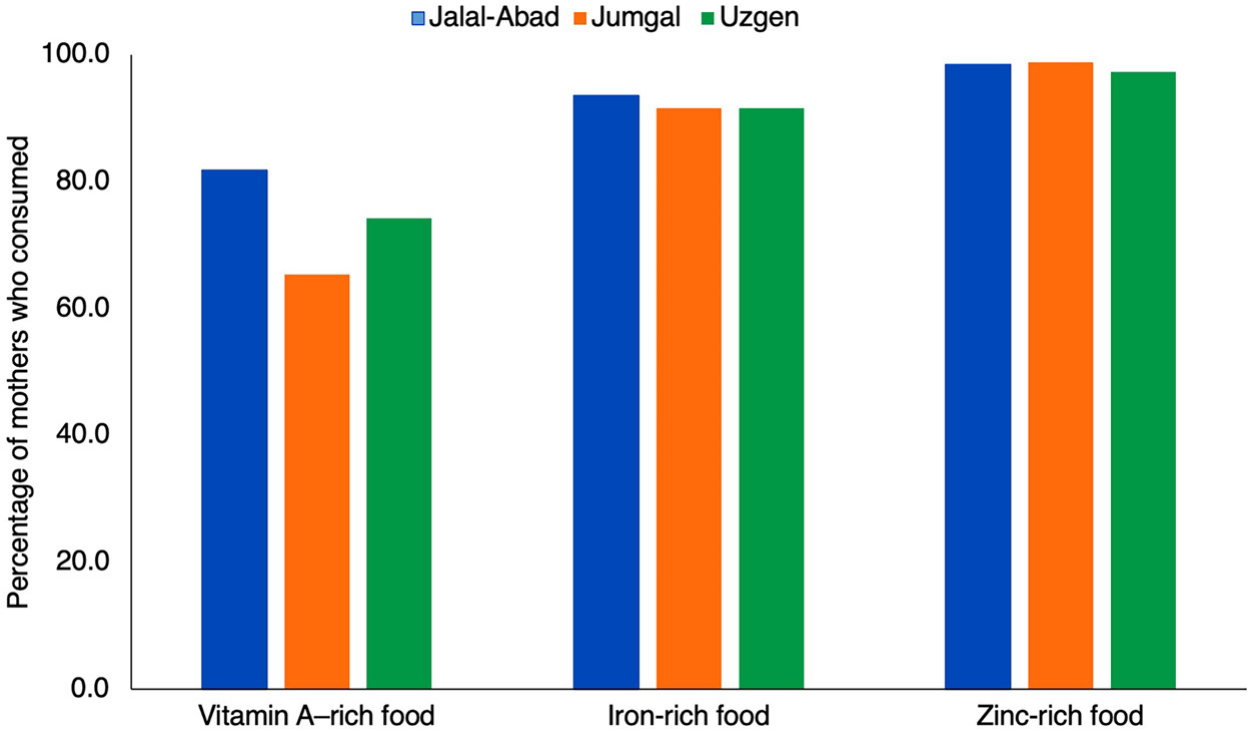
Percentage of Mothers of Children Aged Younger Than 2 Years Who Consumed Micronutrient-Rich Foods, by Region, Kyrgyz Republic

### Source of Foods

Access to food plays an important role in diet diversity. The majority of rural families produce most of their staple food crops (grains and potatoes). Although home gardening is a local custom, production is not high due to labor-intensive traditional techniques.^13^

The survey collected data on the sources, which varied by area, of the micronutrient-rich foods consumed by mothers. We examined the sources of 8 key foods that were rich in vitamin A, iron, and zinc, promoted by the government and SPRING. Of the 8 foods selected, in all 3 areas the majority of mothers consumed 5 of the foods from the same source (either their own farm or the market). Pumpkin, chicken, and yogurt were sourced from their farms or gardens. Mothers in all 3 areas purchased carrots and dark green leafy vegetables from the market. In Jalal-Abad and Uzgen, the majority purchased beef and mutton in the market whereas mothers in Jumgal were more likely to source them from their own farms. Of those who consumed eggs, the majority of mothers reported sourcing their eggs from their own farms/gardens in Jumgal and Uzgen, but in Jalal-Abad, which included mostly rural and some periurban and rural areas, mothers reported being almost equally likely to source eggs from their farms/gardens as from the market (Table 3).

**TABLE 3.** Sources of Foods Among Mothers of Children Aged Younger Than 2 Years Who Consumed 8 Select Foods, by Region, Kyrgyz Republic

**Food**	**Source**	Jalal-Abad, %(95% CI)	Jumgal, %(95% CI)	Uzgen, %(95% CI)
Pumpkin^a^	Own farm/ households	74.1 (59.0, 85.1)	93.3 (67.2, 98.9)	69.1 (57.1, 79.0)
	Market/Shops	25.9 (14.9, 40.9)	6.7 (1.0, 32.8)	30.9 (21.0, 42.0)
Carrots^a^	Own farm/ households	17.1 (10.1, 25.6)	41.2 (31.6, 51.5)	25.1 (15.4, 38.0)
	Market/Shops	82.9 (74.4, 89.1)	58.8 (48.4, 68.9)	74.9 (61.9, 84.6)
Dark green leafy vegetables^a^	Own farm/ households	10.7 (4.3, 24.0)	32.0 (21.7, 44.3)	9.1 (4.1, 19.3)
	Market/Shops	89.3 (75.9, 95.7)	68.0 (55.6, 78.2)	90.9 (80.7, 95.9)
Beef^b^^,^^c^	Own farm/ households	9.5 (4.0, 20.1)	69.2 (59.2, 77.7)	1.6 (0.6, 3.8)
	Market/Shops	90.5 (79.5, 95.8)	30.8 (22.3, 40.8)	98.4 (96.2, 99.0)
Mutton^b^^,^^c^	Own farm/ households	43.8 (32.7,55.5)	94.4 (89.4, 97.0)	34.5 (24.6, 45.8)
	Market/Shops	56.3 (44.5, 67.3)	5.6 (2.9, 10.5)	65.6 (54.2, 75.4)
Chicken^b^^,^^c^	Own farm/ households	66.2 (49.1, 79.8)	78.8 (64.5, 88.3)	94.4 (88.7, 97.3)
	Market/Shops	33.9 (21.1, 50.9)	21.2 (11.6,35.5)	5.6 (2.7, 11.3)
Eggs^c^	Own farm/ households	49.0 (33.8, 64.4)	65.1 (56.7, 72.6)	83.2 (74.1, 89.5)
	Market/Shops	51.0 (35.7, 66.2)	34.9 (27.3, 43.7)	16.8 (10.4, 25.9)
Yogurt /Ayran^c^	Own farm/ households	52.6 (39.7, 66.7)	80.3 (65.3, 89.8)	72.7 (59.4, 82.8)
	Market/Shops	47.5 (33.3, 62.0)	19.7 (10.1, 19.4)	27.3 (17.1, 40.5)

Abbreviation: CI, confidence interval.

a Vitamin A–rich foods.

b Iron-rich foods.

c Zinc-rich foods.

### Food Storage and Preservation

The majority of mothers in all areas reported storing or preserving staple foods (grains, roots, and tubers). Other fruits (apples, pears, persimmons, and melon) were the second most commonly stored or preserved foods. A smaller percentage of women reported preserving vitamin A–rich fruits and vegetables—particularly in Jumgal where few women stored or preserved vitamin A–rich fruits and vegetables (Table 4).

**TABLE 4.** Percentage of Mothers of Children Aged Younger Than 2 Years Who Either Stored or Preserved Select Food Groups, by Region, Kyrgyz Republic

Food Group	Jalal-Abad, %(95% CI)	Jumgal, %(95% CI)	Uzgen, %(95% CI)
Grains and tubers^a^	63.5 (45.5, 78.3)	78.2 (71.5, 83.2)	66.3 (58.6, 72.8)
Vitamin A–rich fruits and vegetables^b^	44.8 (31.9, 54.8)	18.1 (12.5, 25.4)	61.6 (47.6, 73.9)
Other vegetables^c^	55.2 (42.1, 67.5)	43.3 (31.9, 55.3)	64.1 (53.4, 73.5)
Other fruits^d^	55.4 (39.5, 70.2)	57.4 (50.5, 64.1)	61.9 (51.4, 67.9)

Abbreviation: CI, confidence interval.

a Such as oats, barley, potatoes, and turnips.

b Such as pumpkin, carrots, and tomatoes.

c Such as cabbage, onions, eggplant, and cucumbers.

d Such as apples, pears, persimmons, and melon.

Table 5 shows the results of the bivariate analysis of variables that we hypothesized could be associated with MDD-W. Maternal age and education level, whether participants stored or preserved foods, the number of foods stored, whether food remained at the time of the survey, and the region in which they lived were all significant with MDD-W (Table 5). Although important, we were unable to look at the source of foods using bivariate analysis with this data set because sources were asked for individual foods and there was no common indicator.

**TABLE 5.** Minimum Dietary Diversity Among Mothers of Children Aged Younger Than 2 Years, By Background Characteristics and Food Storage and Preservation Practices, Kyrgyz Republic

**Maternal Characteristics**	**Mothers Who Have MDD-W, %**	***P* Value**
Age, years		.004^a^
18–25	63.4	
26–30	73.0	
31–35	69.7	
36–40	64.1	
≥41	84.2	
Education		<.001^b^
≤9 years	58.1	
0–11 years	65.6	
Vocational/technical	74.7	
Attended or completed postsecondary education	75.5	
Household size, No.		.297
1–4	68.0	
5–7	70.3	
≥8	65.3	
Income source		.19
Agriculture (raising livestock or crops)	66.3	
Laborers/migrant workers	68.1	
Business/commerce/salaried professionals	73.4	
Others	67.9	
Smallholding farm/home garden		.913
No	68.7	
Yes	69.0	
Ethnicity		
Kyrgyz	69.0	.512
Tatar/Russian	63.8	
Uzbek	71.2	
Preserved or stored food		.041^c^
No	62.7	
Yes	69.9	
Number of foods stored		
0–1	60.5	<.001^b^
2–3	67.7	
≥4	81.6	
Leftover food from store or preserved		<.001^b^
No	61.1	
Yes	71.6	
Region		<.001^b^
Uzgen	68.1	
Jumgal	62.4	
Jalal-Abad	76.0	

Abbreviation: MDD-W, minimum diet diversity for women.

a Statistically significant *P*<.01.

b Statistically significant *P*<.001.

c Statistically significant *P*<.05.

According to the logistic regression, having some postsecondary education was nearly but not statistically significant (odds ratio [OR]=1.69; 95% confidence interval [CI]=0.98, 2.90). Contrary to expectation, mothers with smallholding farms or gardens were less likely to achieve MDD-W (OR=0.74; 95% CI=0.56, 0.98). Those who had stored 4 or more foods during winter were 2.44 times more likely (95% CI=1.62, 3.65) to consume a diverse diet than those who did not. Those with preserved foods remaining at the time of the survey were 1.62 times more likely (95% CI=1.23, 2.14) to have consumed a diverse diet. Although those who lived in Jalal-Abad were 1.4 times more likely (95% CI=1.00, 1.95) to consume a diverse diet than those who lived in Uzgen; this was not statistically significant. However, those who lived in Jumgal were less likely to consume a diverse diet (OR=0.71; 95% CI=0.51, 1.00; *P*<.05) than those who lived in Uzgen (Table 6).

**TABLE 6.** Determinants of Maternal Dietary Diversity, Kyrgyz Republic

**Maternal Dietary Diversity**	**Odds Ratio**	**95% CI**	***P* Value**
Age, years	1.02	1.00, 1.04	.108
Household size	0.96	0.90, 1.02	.207
Education			
≤9 years	1 [Reference]		
10–11 years	1.13	0.69, 1.85	.618
Vocational or technical	1.61	0.88, 2.95	.120
At least some higher education	1.69	0.98, 2.90	.058
Owns or rents smallholding, or gardens			
No	0 [Reference]		
Yes	0.74	0.56, 0.98	.034^a^
Household source of income			
Raising, selling livestock	1 [Reference]		
Raising, selling crops	0.93	0.66, 1.29	.656
Salaried labor, construction, paid farm, business	1.13	0.80, 1.61	.476
Other	1.25	0.87, 1.80	.220
Ethnicity			
Kyrgyz	1 [Reference]		
Tatar/Russian	0.89	0.51, 1.56	.677
Uzbek	0.90	0.54, 1.50	.691
Number of foods stored			
0–1	1 [Reference]		
2–3	1.30	0.93, 1.81	.122
≥4	2.44	1.62, 3.65	<.001^b^
Stored food remaining			
No	1 [Reference]		
Yes	1.18	0.83, 1.66	.356
Preserved food remaining			
No	1 [Reference]		
Yes	1.62	1.23, 2.14	.001^c^
Region			
Uzgen	1 [Reference]		
Jumgal	0.71	0.51, 1.00	.048^a^
Jalal-Abad	1.40	1.00, 1.95	.052
Constant	0.76	0.34, 1.70	.505

Abbreviation: CI, confidence interval.

a *P*<.05.

b *P*<.001.

c *P*<.01.

## DISCUSSION

Despite expected challenges to achieving a diverse diet in winter, most mothers in the survey population consumed 5 or more of the 10 MDD-W food groups, indicating a greater likelihood of consuming a micronutrient-adequate diet than if they ate fewer food groups. As would be expected, after controlling for several maternal and household characteristics, preserving enough food to last through winter and storing a variety of foods were both associated with maternal dietary diversity. Access to a home garden was not associated with higher odds of consuming minimum diet diversity. Living in more urban areas of Jalal-Abad was associated with an increased likelihood of consuming an MDD-W, likely due to easy access to markets. Muthini et al. found in Kenya that farm production has a small effect on dietary diversity but a more negative impact on diet diversity from markets.^27^ Other studies have also suggested a positive association between access to markets and dietary diversity.^28^^,^^29^ As we see in our study but could not measure, markets are an important source of micronutrient-rich foods, and mothers in all 3 areas had access to them. However, living in Jumgal appears to have a negative association with dietary diversity, with the lowest percentage of mothers consuming vitamin A–rich foods and eggs there despite having the highest percentage of home gardens. This area is remote, has limited accessibility in winter due to harsh weather conditions, and is located in Naryn, one of the poorest oblasts.^13^^,^^14^ Economic constraints are often a barrier to purchasing foods in local markets, along with weather conditions that make travel to markets in parts of Jumgal prohibitive.

Most mothers in the survey population consumed 5 or more of the 10 MDD-W food groups.

In the Kyrgyz Republic, where seasonality affects food availability, the government and SPRING built on culturally appropriate home processing and storage practices to promote consumption of a variety of nutrient-dense foods to mitigate some of the physical constraints to improved diet diversity in winter. Our results suggest markets may also play an important role in improving diet diversity. In this setting, to increase the prevalence of quality maternal diets, it is important to understand and address the sociocultural, structural, and/or physical barriers and constraints to market access along with women’s ability to store and preserve foods in vulnerable regions of the country.

### Limitations

The limitations of this study are primarily based on the nature of secondary data analysis. Due to how some data were coded, we were unable to construct a market variable to explore this issue. We do not have information on the quantities of food consumed and therefore cannot say whether the mothers were getting adequate amounts of food or if their consumption of meats and other animal-source foods are within healthy limits. We also have no information on nutritional status to know how many mothers in the sample were undernourished, overweight, or obese. We also have no information on the consumption of highly processed foods, or whether fats, salts, and sugars were added during home preservation and storage. The data were limited to a small number of rayons in each of 3 oblasts, and the findings should not be extrapolated to other areas of the Kyrgyz Republic. The analysis can only look at associations, and no inference is being made to causality.

## CONCLUSION

In addition to micronutrient adequacy, high-quality diets are characterized by adequate consumption of healthy foods and limited consumption of unhealthy, low-nutrient foods (i.e., highly processed foods, saturated fats, sugar, and sodium) associated with increased risk for noncommunicable diseases.^30^ The findings of our study are encouraging; during a season when one expects more food insecurity, even in remote rural areas, mothers in the Kyrgyz Republic are able to access a variety of foods and consume a diverse diet. Diet diversity was strongly associated with cultural practices of preserving and storing foods in the fall, particularly storing a variety of foods (more than 5) and preserving enough food to last through winter. The government and programs should build on these findings and further explore any barriers women face to storing, preserving, and accessing vitamin-rich foods in markets. Although we did not look at consumption of unhealthy and low-nutrient foods, given the rates of overweight and obesity among women, interventions should also explore culturally acceptable ways to store and preserve foods without increasing the consumption of empty calories with the increased risk of overweight, obesity, or noncommunicable diseases.

## Notes

**Peer Reviewed**

**First published online**: November 11, 2022.
